# Mechanical properties analysis of the interspersed replacement of timber sleepers with bamboo-based composite sleepers on open bridge deck

**DOI:** 10.1038/s41598-025-28871-0

**Published:** 2025-12-06

**Authors:** Peng Chen, Tiexu Liu, Wang Xu, Jiajun Huang, Chenghui Li

**Affiliations:** 1 Beijing Urban Construction Design & Development Group Co., Ltd. , Beijing, 100037 China; 2 National Engineering Research Center of Green & Safe Construction Technology in Urban Rail Transit, Beijing, 100037 China; 3 Chengdu Southwest Jiaoda High-speed Railway Equipment Co., Ltd. , Chengdu, 610031 China; 4 MOE Key Laboratory of High-Speed Railway Engineering, Southwest Jiaotong University , Chengdu, 610031 China

**Keywords:** Bamboo-based composite sleeper, Open bridge deck, Dynamic characteristics, Timber sleeper, Steel truss bridge, Engineering, Materials science

## Abstract

Due to the discrete deterioration characteristics of timber sleepers on open bridge deck, continuous replacement is difficult. New bamboo-based composite sleepers (BCSs) offer excellent performance at a low price, making them an ideal alternative to timber sleepers. Therefore, this study focuses on the mechanical properties and safety of the interspersed replacement of timber Sleepers with BCSs. A static model of the open deck track and a vehicle-track coupling dynamic model were established to precisely analyze the influence of 1 in 2, 1 in 3, and 1 in 4 interspersed replacement, as well as full replacement with BCSs on the force and deformation of the track structure and the dynamic characteristics of the vehicle-track system. After the interspersed replacement of timber sleepers with BCSs, the sleeper compression, sleeper vertical displacement, and gauge reduction were decreased; however, the sleeper bending moment, vehicle acceleration, wheel-rail force, and bridge deck acceleration were slightly increased. At the longitudinal beam support, the BCSs showed a negative bending moment, with compression at the bottom and tension at the top. The maximum tensile and compressive stresses were 4.87 MPa and 7.97 MPa, respectively, which are both below the allowable stress of the material—meeting the strength requirements for BCSs. Although the interspersed replacement of timber sleepers with BCSs leads to uneven track stiffness, the track deformation, stress distribution and dynamic response remain within acceptable limits. This research provides new ideas for track structure upgrades and maintenance strategies.

## Introduction

With the high-density operation of railways in China, the performance of track structure components faces severe challenges. Track structures enabled through new technologies and new materials are continuously being developed and applied. At present, some steel truss bridges still use timber sleepers on open bridge deck. Although timber sleepers have advantages such as low weight, favorable elasticity, and ease of installation, they are prone to cracking, decay, and lateral resistance attenuation, which increases the safety risks and maintenance costs of the line^1,2^. In addition, there are issues such as the shortage of high-quality timber resources and anti-corrosion pollution. There is an urgent need to identify new types of sleepers to gradually replace the deteriorated timber sleepers. In recent years, driven by the “Dual Carbon Strategy” (referring to carbon peak and carbon neutrality), composite sleepers made of various environmentally friendly and waste composite materials^3,4^ have been regarded as ideal substitutes for timber sleepers. This is due to their high strength, low weight, strong weather resistance, and low-carbon emissions throughout their life cycle.

At present, composite sleepers are mainly divided into recycled composite sleepers, fiber composite sleepers, BCSs, etc. according to material composition^5^. Recycled composite sleepers are mainly made of waste tires, plastics and other difficult-to-degrade polymer waste as raw materials, supplemented by chemical additives^6,7^; fiber composite sleepers are mainly made from resin as the matrix, mixed with glass fiber, carbon fiber and other reinforcing materials, and formed by molding, pultrusion and other processes^2^; BCSs are made of bamboo as the raw material, maintaining the original arrangement of bamboo fibers, and are pressed after carbonization and other treatments^8^. Currently, recycled composite sleepers and fiber composite sleepers are used in the United States, Europe, China and other countries, and there are many studies on their performance. These studies mainly involve the interaction between composite sleepers and fasteners^9–11^, the longitudinal and transverse resistance of the ballast bed^12^, composite sleeper-ballast contact forces^13^, vibration reduction characteristics^14,15^, bending performance^16–18^, impact resistance^19^, and bearing capacity^3,20^. however, the strength of recycled composite sleepers needs to be improved; fiber composite sleepers exhibit better performance but are more expensive.

Compared with recycled composite and fiber composite sleepers, BCSs are relatively new and have only been laid in test sections. Research on BCSs has mainly focused on processing technology and mechanical properties^21^. Nkeuwa^22^ provided a comprehensive review of bamboo-based composites, emphasizing their bonding behavior, durability, and applicability in railway infrastructure. Xiao^23^ used wood particles and bamboo strips as reinforcing materials and phenol formaldehyde resin as the matrix to manufacture wood-bamboo hybrid composite sleepers. The mechanical properties of these sleepers were tested using various parameters, and the optimal processing parameters were recommended. Jing^8^ investigated the mechanical performance and dynamic characteristics of BCSs through experiments and simulations, further confirming their potential as replacements for timber sleepers. Zhao^6,24^ tested the mechanical properties of BCSs, established a vehicle-track coupling dynamics model, and used the power flow method to analyze the vibration reduction characteristics of BCSs. Based on these analyses, optimal parameters were recommended. BCSs are inexpensive and have good mechanical properties and great application potential.

With the urgent need to replace timber sleepers on open bridge deck, many scholars have conducted research on potential substitutes for timber sleepers. Zhang^25^ evaluated the applicability of glass fiber-reinforced resin composite sleepers on steel truss bridges. The results indicate that applying these composite sleepers ensures the safe operation of trains and keeps the stress and deformation of the track structure within acceptable limits. Sun^26^ conducted full-scale tests and finite element simulation analysis to explore the load distribution mechanism of a new type of steel sleeper on open bridge deck. At present, there is no optimal solution for replacing timber sleepers on open bridge deck.

In view of the urgent need to replace timber sleepers on open bridge deck and the application potential of BCSs, using BCSs to replace deteriorated timber sleepers is an option. In practice, the deterioration of timber sleepers is spatially and temporally dispersed, so their replacement will be implemented on a staggered basis. Due to the differences in stiffness parameters between timber sleepers and BCSs, there may be problems with uneven track stiffness^27–29^ and abnormal dynamic responses of the vehicle-track system under train loads^30^.

Currently, the application of the interspersed replacement of timber sleepers with BCSs on open bridge deck has not been investigated. In order to analyze the feasibility of the interspersed replacement of timber sleepers, this paper establishes a static model of the open bridge deck track and a vehicle-track-bridge coupled dynamic model. It systematically investigates the effects of the interspersed replacement of timber sleepers with BCSs on track deformation and forces, track structure and bridge dynamic characteristics. This study offers a solution that balances mechanical properties and sustainability for the discrete replacement of timber sleepers. It provides a theoretical foundation for promoting and applying BCSs and introduces new concepts for track structure upgrades and full-life cycle maintenance strategies.

## Mechanical model and calculation conditions

### Static analysis model

**Fig. 1:**
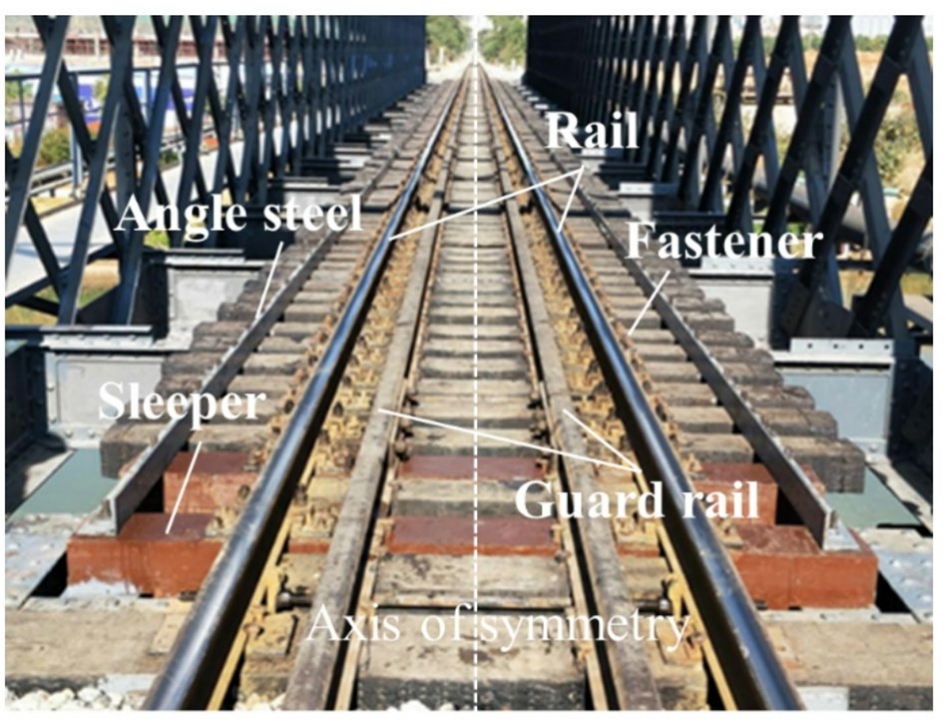
Open bridge deck symmetrical track structure.

**Fig. 2:**
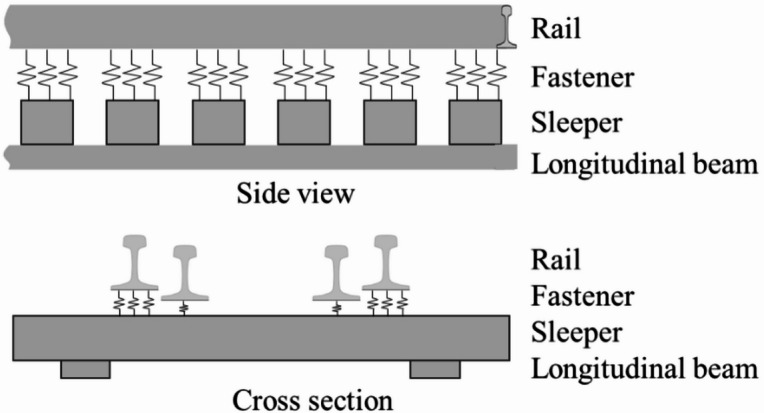
Static model.

This paper used ANSYS software to model and calculate a 64-meter-span, single-line steel truss bridge in an urban rail transit system. The symmetrical track structure on the open bridge deck is composed of CHN60 rail, timber sleepers/BCSs, type II split elastic fastener system, and guard rail, as shown in Fig. 1. The upper parts of the sleepers are connected to the rail through the fastener systems, and the lower parts directly act on the two longitudinal beams. The static analysis model of the open bridge deck track focuses on analyzing the influence of different operating conditions of the interspersed replacement of timber sleepers on the deformation and stress of the track structure. In the model, the rail, sleepers, and longitudinal beams are simulated by solid elements; the fastener system is simplified to spring-damper elements; and the guard rail are simplified to Timoshenko beam elements. The static model is shown in Fig. 2. To eliminate boundary effects and reduce computational cost, the model length is set to 22 sleeper spans, with the train load applied in the middle. The load is set to be 16 tons for the A-type train axle, with a speed of 80 km/h. The dynamic load coefficient is $a=0.6V/100=0.48$, and the single wheel load of the calculated load is 118.4 kN. The bottom layer of the longitudinal beam and both ends of the rails are constrained. The other parameters of the static model refer to reference^24^ and test report values, as shown in Table 1.

**Table 1:** The parameters of the track.

Parameters		Unit	Value
Rail	Elastic modulus	N/m^2^	2.06 × 10^11^
	Density	kg/m^3^	7800
	Mass per unit length	kg/m	60.64
	Poisson ratio	-	0.3
Fastener	Vertical stiffness	N/m	5.0 × 10^7^
	Vertical damping	N·s/m	7.5 × 10^4^
Sleeper	Sleeper spacing	m	0.44
	Length/width/height	m	2.6/0.22/0.16
	Poisson ratio	-	0.2
BCS	Elastic modulus	N/m^2^	2.5 × 10^10^
	Density	kg/m^3^	1200
Timber sleeper	Elastic modulus	N/m^2^	1.23 × 10^10^
	Density	kg/m^3^	1250
Longitudinal beam	Elastic modulus	N/m^2^	2.06 × 10^11^
	Density	kg/m^3^	7800
	Poisson ratio	-	0.3
	Spacing	m	2.02
	Width/height	m	0.22/0.16

### Dynamic analysis model

To analyze the dynamic characteristics of the track structure, a vehicle-track coupling dynamic model^31^ was also established. In the model, the vehicle is simplified to a 10-degree-of-freedom system, considering the vertical and pitch motion of the car body and bogie, and the vertical motion of the wheelset. The primary suspension and the secondary suspension are modeled as spring-damper elements.

**Fig. 3:**
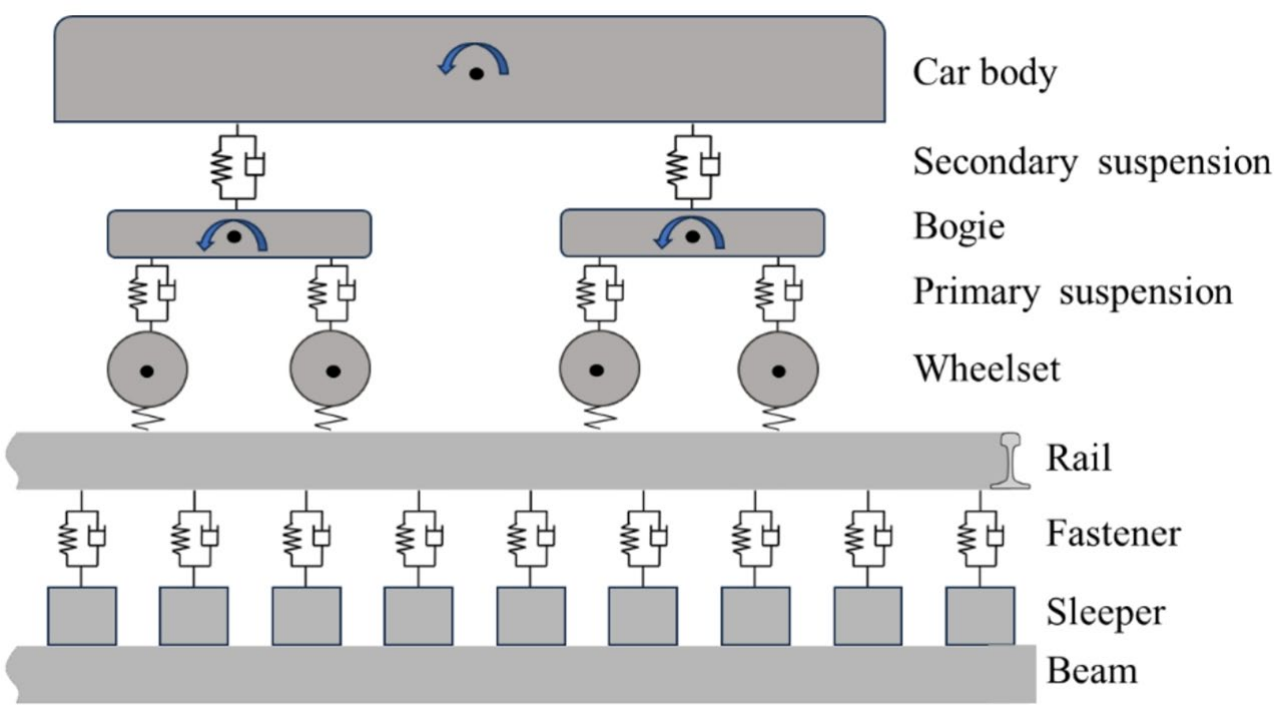
Vehicle-track coupling dynamic model.

In the track model, the rail is simplified to a point-supported Euler beam, the sleepers are simulated by solid elements, the fasteners are modeled as spring-damper elements, and the bridge is simplified to plate elements. The dynamic model is shown in Fig. 3.

The bridge described is a standard design for a railway steel truss bridge. The bridge span is 64 m, with eight bays. The main truss height is 11 m and the bridge width is 5.75 m. The bridge’s moment of inertia is primarily considered from the upper and lower chords. Both the upper and lower chords are I-shaped. The upper chord’s web measures 0.412 × 0.016 m, with a flange of 0.46 × 0.024 m. The lower chord’s web measures 0.42 × 0.012 m, with a flange of 0.46 × 0.02 m. Calculation shows that the moment of inertia is 3.1252 m^4^. Simplifying it to a plate element, the equivalent moment of inertia is I = bh^3^/12, where b and h are the width and height, respectively. Assuming the width b is the sleeper length of 2.6 m, h = 2.43 m.

The vehicle and track are coupled through wheel-rail interaction. The nonlinear Hertzian elastic contact theory^32^ is used to calculate the wheel–rail force. Therefore, the wheel–rail force is simulated by Eqs. 1, 2:

1 $$\left\{ \begin{gathered} {[\frac{1}{{{G_{Hertz}}}}\Delta Z(t)]^{3/2}},\Delta Z(t)>0 \hfill \\ 0,{\text{ }}\Delta Z(t) \leqslant 0 \hfill \\ \end{gathered} \right.$$

2 $$\Delta Z(t)={Z_w}(t) - {Z_r}(t) - \eta (t)$$

where ${G_{Hertz}}$ is the contact constant of wheel and rail (m/N^2/3^), $\Delta Z(t)$ is the normal compression at the wheel–rail contact point, ${Z_w}(t)$ is the vertical displacement of the wheel, ${Z_r}(t)$ is the vertical displacement of the rail at the relevant contact point, and $\eta (t)$ is the track vertical irregularity.

The American five-level spectrum is utilized as the irregularity excitation in the model. The vehicle model adopts an A-type train running at 80 km/h, and the vehicle parameters^33^ are shown in Table 2.

**Table 2:** The main parameters of the A-type train.

Name	Unit	Value
Mass of car body	kg	42 507
Mass of bogie	kg	2 721
Mass of wheelset	kg	1 900
Pitching moment of inertia of car	kg·m^2^	1.94 × 10^6^
Pitching moment of inertia of bogie	kg·m^2^	1752
Vertical stiffness of primary suspension	N/m	0.75 × 10^6^
Vertical stiffness of secondary suspension	N/m	1.349 × 10^6^
Vertical damping of primary suspension	N·s/m	1.96 × 10^4^
Vertical damping of secondary suspension	N·s/m	4.0 × 10^4^
Length, width, and height of car body	m	22/3/3.8
Fixed axle spacing	m	2.5
Bogie center distance	m	15.7
Wheel radius	m	0.42

### Model validation

**Fig. 4:**
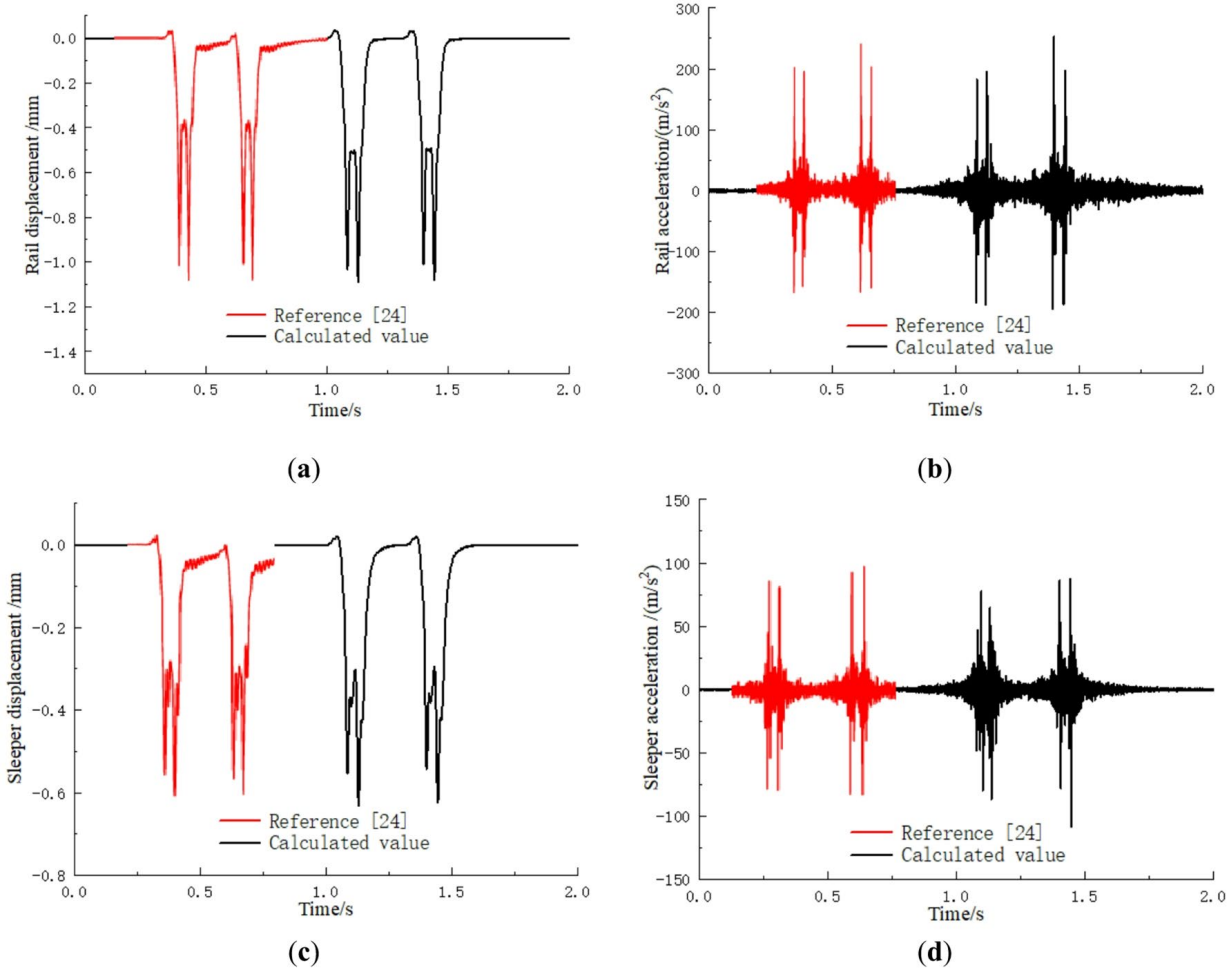
Model validation: (**a**) Rail displacement; (**b**) Rail acceleration; (**c**) Sleeper displacement; (**d**) Sleeper acceleration.

To verify the validity of the models, the parameters from reference^24^ were substituted into the model proposed in this paper for calculation. Figure 4 shows that the displacement and acceleration time history curves in this paper are consistent with those in reference^24^. Table 3 shows that the peak displacement and acceleration values are also close to the values calculated in reference^24^. This demonstrates the accuracy of the model.

**Table 3:** The maximum responses.

Types	Responses	Zhao^24^	This paper	Error/%
Static analysis	Sleeper displacement/mm	0.156	0.161	3.21
	Sleeper compression/mm	0.027	0.0285	5.55
Dynamic analysis	Rail displacement/mm	1.05	1.09	3.81
	Sleeper displacement/mm	0.60	0.63	5.00
	Rail acceleration/(m/s^2^)	236.8	253.5	7.05
	Sleeper acceleration/(m/s^2^)	97.1	108.2	11.43

### Calculation conditions

To compare and analyze the effects of different levels of BCS interspersion in the timber sleeper system, different operating conditions were used: all timber sleepers, all BCSs, 1 in 2, 1 in 3, and 1 in 4. The “1 in n” means that there is one BCS in every n sleepers. Additionally, to analyze the role of the guard rail in the track structure, models with and without guard rail were also established.

## Results and discussion

### Track deformation and force

#### Rail deformation

**Fig. 5:**
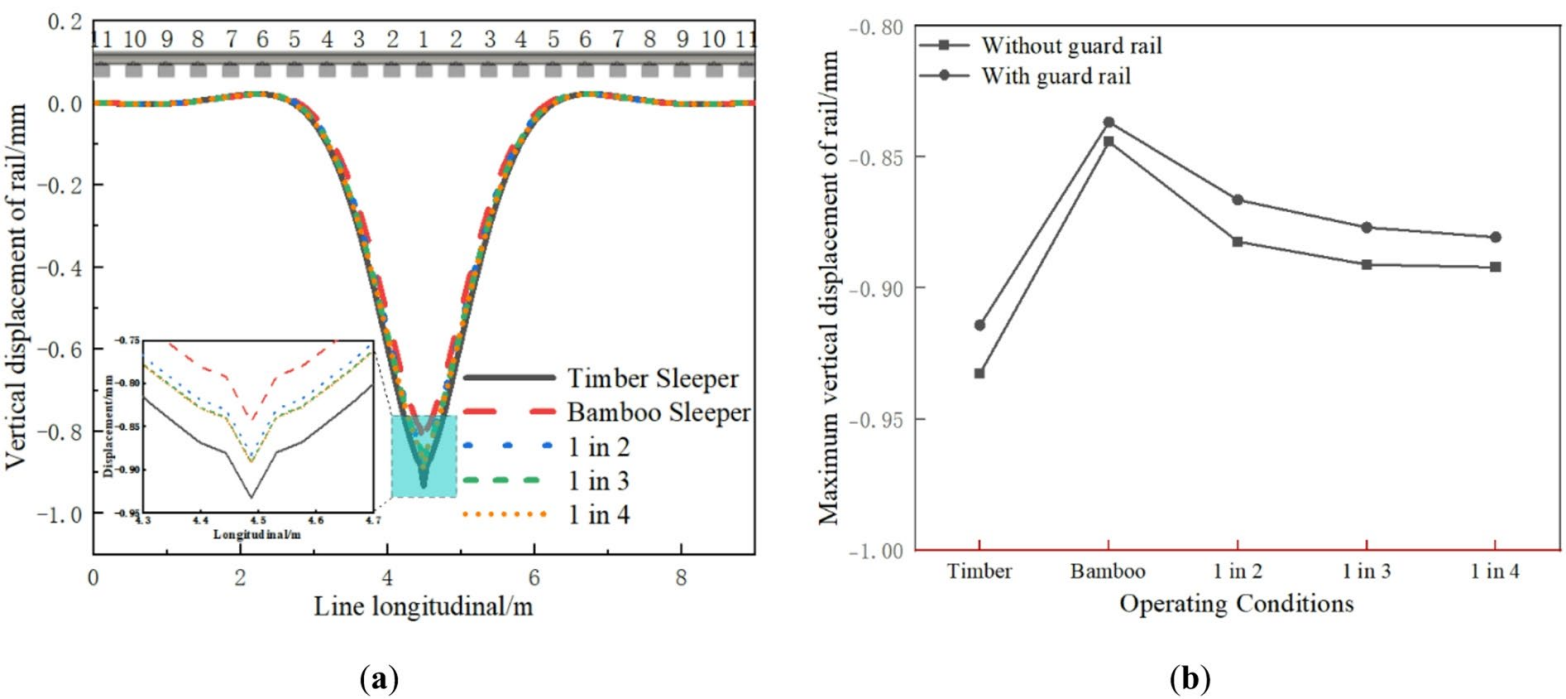
Vertical displacement of rail: (**a**) Longitudinal distribution; (**b**) Maximum value comparison.

As shown in Fig. 5a, under different operating conditions (all without guard rail), the wheel load is distributed longitudinally and symmetrically across 7 sleepers. The vertical displacement of the rail near the wheel load is the largest, and the load borne by the sleeper is also greater. The timber sleeper track shows the largest vertical rail displacement, while the rail displacement decreases in sequence for the 1 in 4, 1 in 3, 1 in 2, and BCSs tracks by approximately 4.34%, 4.46%, 5.39%, and 9.48%, respectively. This indicates that as the number of interspersed BCSs increases, the overall track stiffness improves, reducing the vertical displacement of the rail. As shown in Fig. 5b, adding guard rail can further enhance the stability and stiffness of the track. The effect is most pronounced for the timber sleepers track, and the reduction in vertical rail displacement is the largest.

**Fig. 6:**
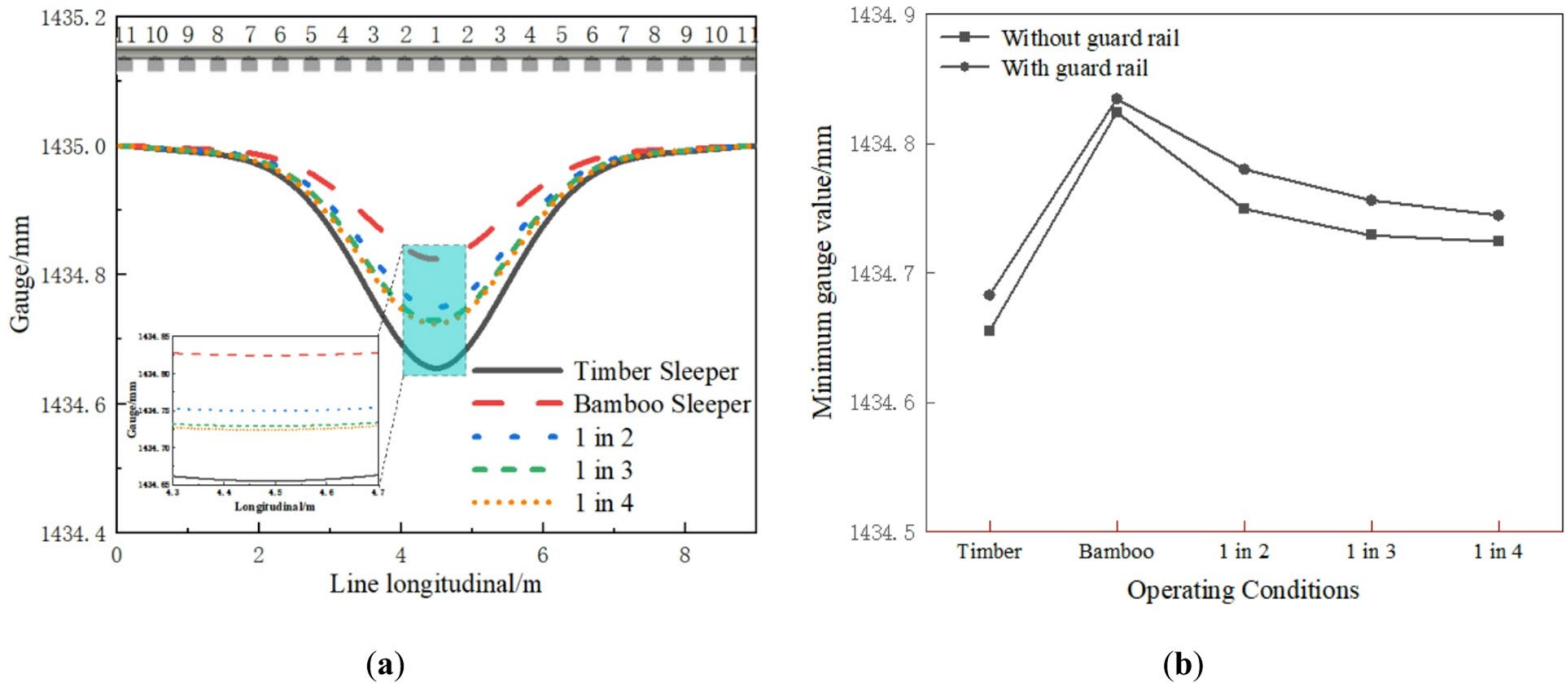
Track gauge variation: (**a**) Longitudinal distribution; (**b**) Maximum value comparison.

Under wheel load, the sleepers exhibit uneven longitudinal deformation, which can affect the track geometry. Figure 6 illustrates the track gauge changes under different operating conditions. As shown in Fig. 6a, the influence range of track gauge under different operating conditions is about the span of 9 sleepers. The gauge change of the timber sleepers track is slightly larger, and the gauge change of the BCSs track is the smallest. Overall, the gauge reductions under different operating conditions are small and remain well below the 2 mm limit stipulated in the “Code for Construction and Acceptance of Urban Rail Transit Projects” (GB 50490 − 2009). As shown in Fig. 6b, adding guard rail can further reduce the track gauge change, which is beneficial for maintaining track geometry.

#### Sleeper deformation and bending moment

**Fig. 7:**
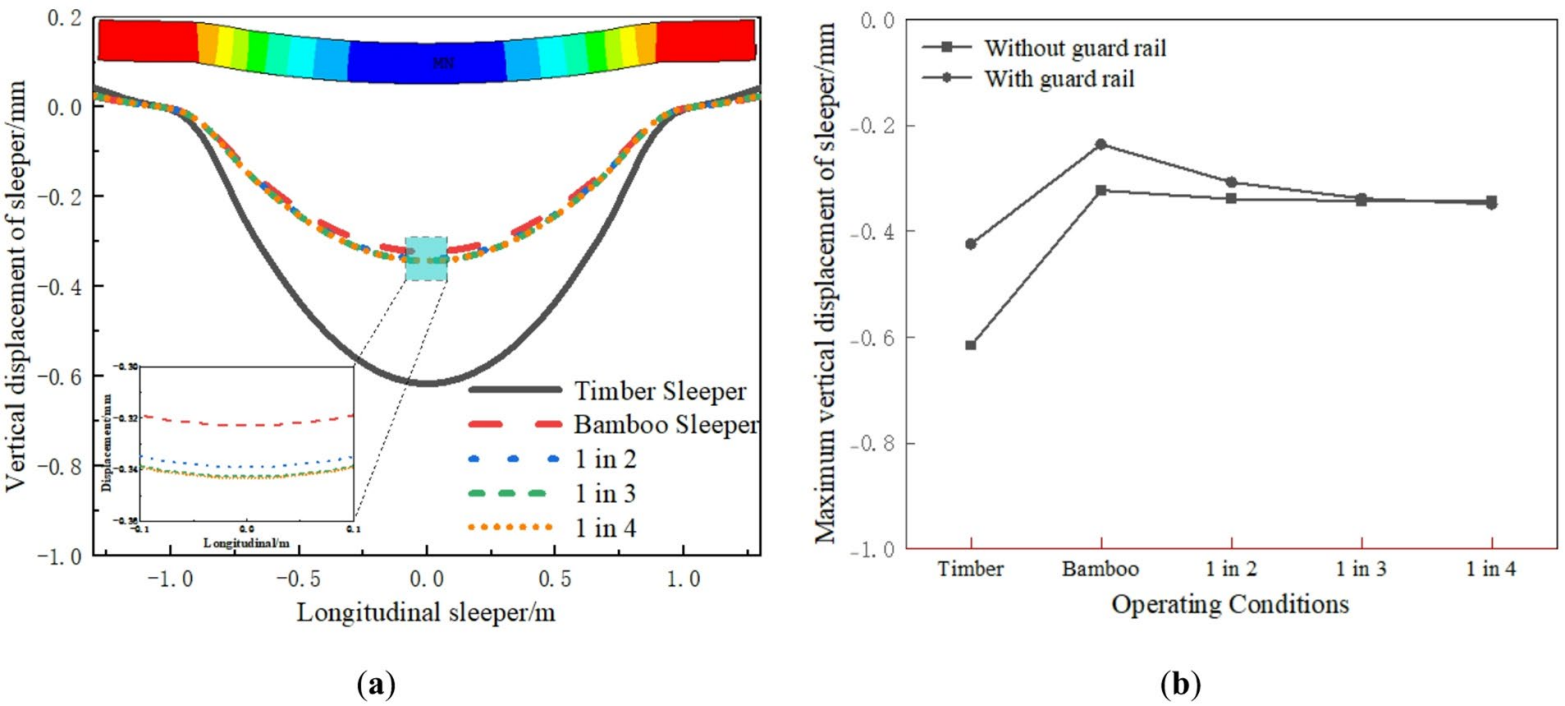
Vertical displacement of sleeper: (**a**) Longitudinal distribution; (**b**) Maximum value comparison.

The vertical displacement of sleepers under different operating conditions is shown in Fig. 7. Relative to the rail, the two longitudinal beams are supported symmetrically outside the sleepers. The maximum vertical displacement of the sleeper occurs in the middle, while the end exhibits slight deflection. The vertical displacement of the timber sleepers track is the largest, measuring 0.62 mm. The sleeper displacements of the 1 in 4, 1 in 3, 1 in 2 and BCSs track are significantly lower, showing reductions of approximately 44.32%, 44.41%, 45.01%, and 47.63%, respectively. The interspersed BCSs greatly improves the bending stiffness and significantly reduces the bending deformation of the sleeper. As shown in Fig. 7b, adding guard rail significantly reduces the maximum vertical displacement of all timber sleepers and all BCSs, but it has a minimal effect on reducing the displacement of the interspersed sleepers.

**Fig. 8:**
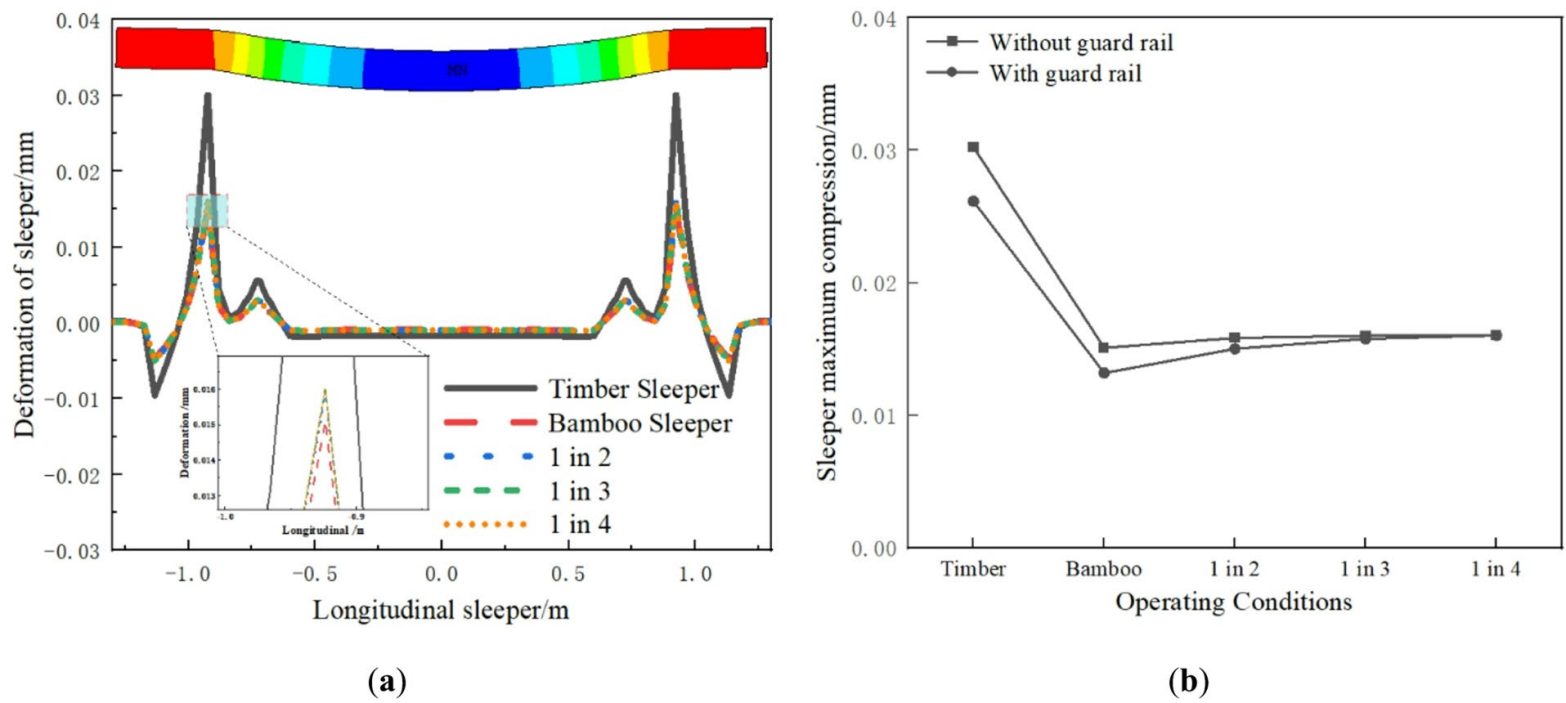
Deformation of sleeper: (**a**) Longitudinal distribution; (**b**) Maximum value comparison.

Under wheel load, the sleeper not only exhibits bending deformation, but also symmetrical compression deformation in the cross section. As shown in Fig. 8a, the compression deformation of the sleeper is largest at the longitudinal beam support, followed by at the rail seats. The sleeper cross section outside the longitudinal beam exhibits tensile deformation. The main reason is that the sleeper’s middle undergoes downward bending, creating an inflection point at the inner support of the longitudinal beam. Additionally, upward bending occurs on both sides of the sleeper. The longitudinal beam’s constraint causes tensile deformation in the sleeper cross section outside the beam. The compression of the timber sleeper is the largest, at approximately 0.03 mm. In other operating conditions, the sleeper compression is significantly reduced. As shown in Fig. 8b, the guard rail reduces timber sleeper compression but has a minimal effect in other conditions.

**Fig. 9:**
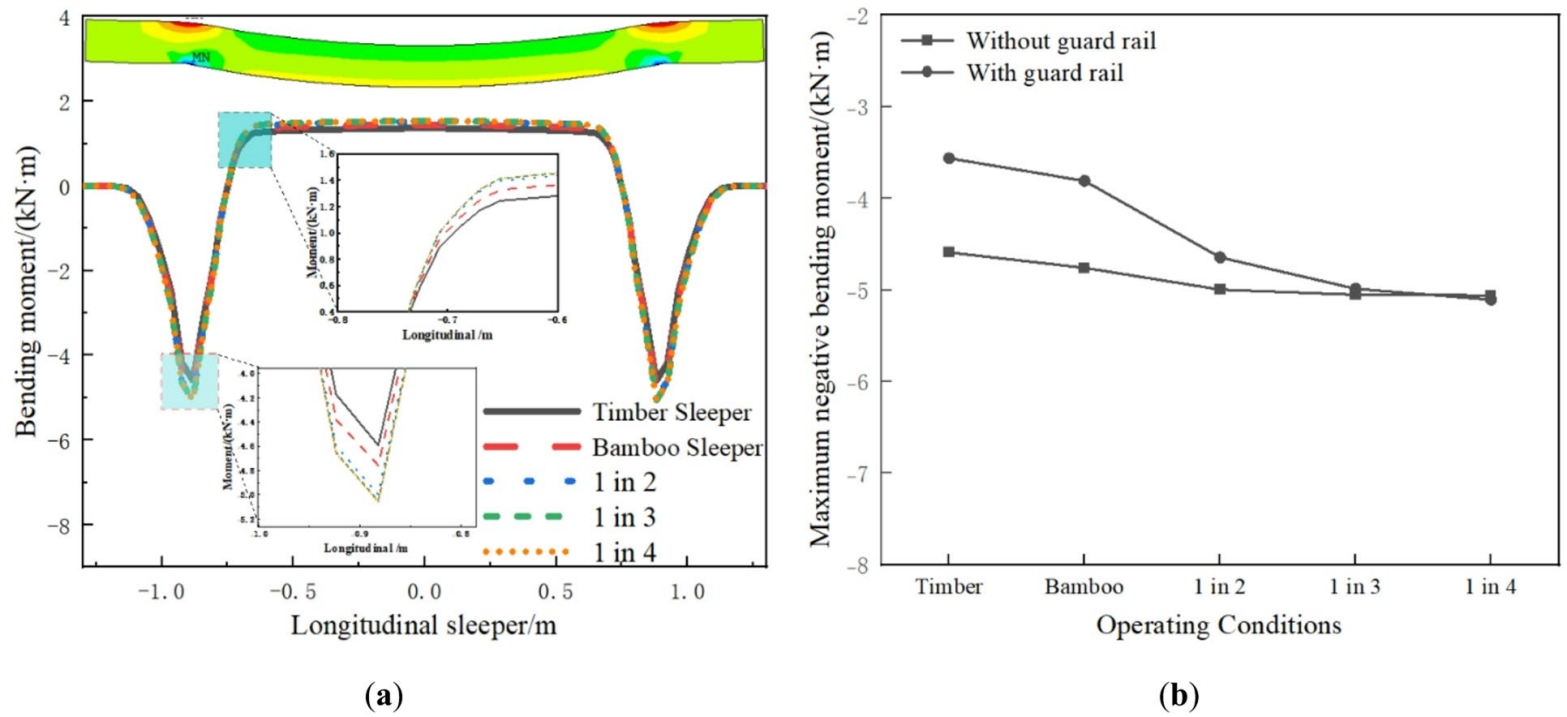
Sleeper bending moment: (**a**) Longitudinal distribution; (**b**) Maximum value comparison.

The longitudinal bending moment of the sleeper is shown in Fig. 9. At the longitudinal beam support, the sleeper exhibits a negative bending moment, while the middle part shows a positive bending moment. The bending moment distribution of the sleeper differs significantly from that of a traditional four-point supported long beam. The main reason for this is that the sleepers in the model are solid elements, and the internal deformation of the sleeper structure is considered. The sleeper and the longitudinal beam are in surface contact, not point contact, and in practice, the longitudinal beam and the sleeper are constrained by bolts. The model simulates the real situation through coupling. Therefore, under the train load, the lower part of the sleeper is longitudinally compressed and the upper part is longitudinally tensile at the longitudinal beam support, resulting in a negative bending moment. This is consistent with the experimental conclusions of the composite sleepers in reference^34,35^. In reference^34^, when the train load is applied to the composite sleepers, the strain at the sleeper support shows longitudinal compression at the lower part and longitudinal tension at the upper part. In reference^35^, the bending moment of the sleeper with five-point support also shows a significant negative bending moment at the middle support. This also confirms the accuracy of the model in this paper. The bending moment of the timber sleeper track is slightly smaller, followed by the BCSs track. The bending moments of the three interspersed sleepers tracks are relatively close and slightly larger than the timber sleepers and the BCSs tracks. Under different operating conditions, the maximum tensile and compressive stresses of the BCSs are 4.87 MPa and 7.97 MPa, respectively, both below allowable values, ensuring the safety of the BCSs. As shown in Fig. 9b, the guard rail reduces the bending moment of the timber sleepers and the BCSs.

### Dynamic applicability

#### Vehicle acceleration

**Fig. 10:**
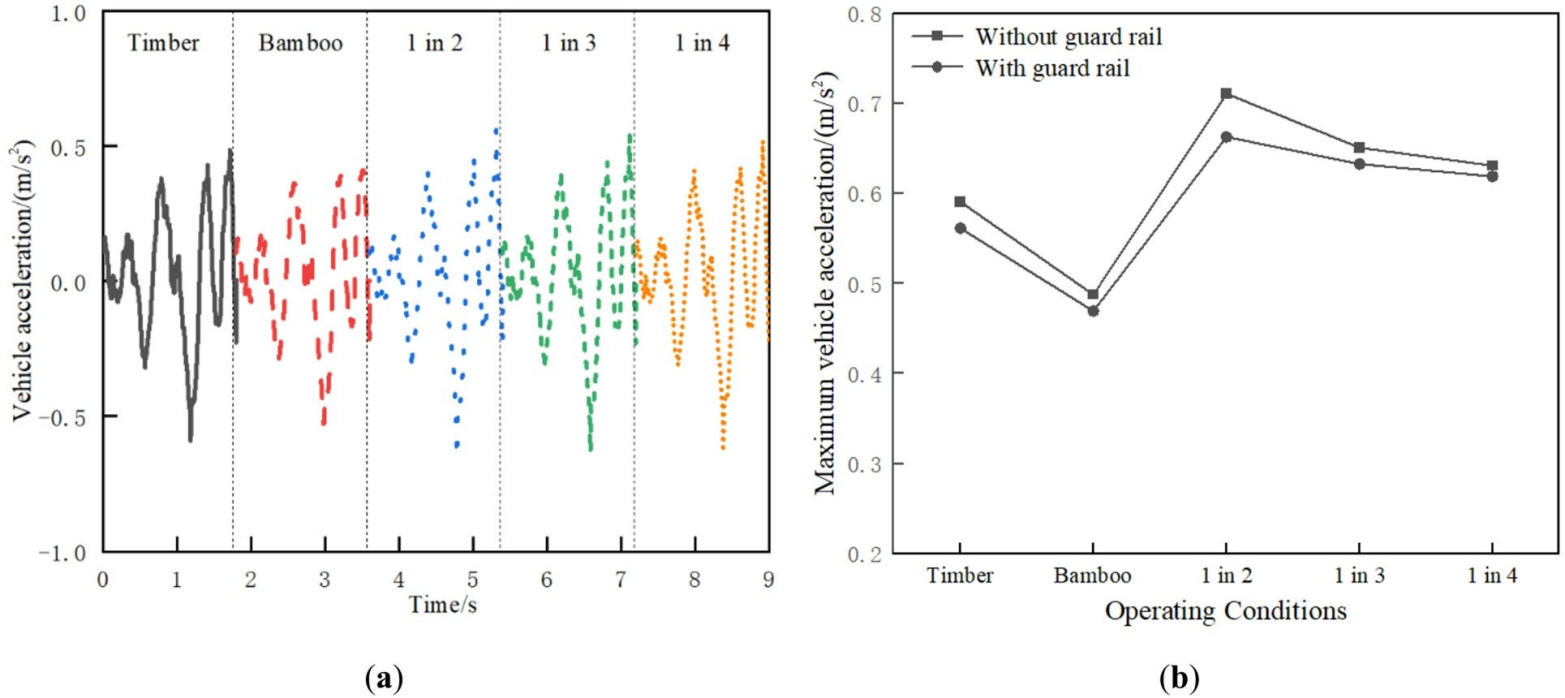
Vehicle acceleration: (**a**) Time history curve; (**b**) Maximum value comparison.

In order to clearly compare and analyze the time history curves of the structural dynamic response under different operating conditions, the time history curves in the following figures each place the dynamic responses of different operating conditions on the continuous time axis. Based on the dynamic model, the vehicle acceleration under different operating conditions is shown in Fig. 10. As shown in Fig. 10a, the vehicle acceleration for 1 in 2 interspersed track is the largest at 0.64 m/s², which is well below the threshold of 2.5 m/s². The accelerations of 1 in 3, 1 in 4, all timber sleepers, and all BCSs tracks decrease sequentially, showing reductions of approximately 1.55%, 2.97%, 7.79%, and 17.64%, respectively. Although the vehicle accelerations under different operating conditions meet the specification requirements, the interspersed replacement of timber sleepers causes uneven track stiffness, increasing the vehicle vibration acceleration. As shown in Fig. 10b, adding guard rail reduces vehicle acceleration, with the most significant reduction observed in the 1 in 2 interspersed condition.

#### Wheel-rail force

**Fig. 11:**
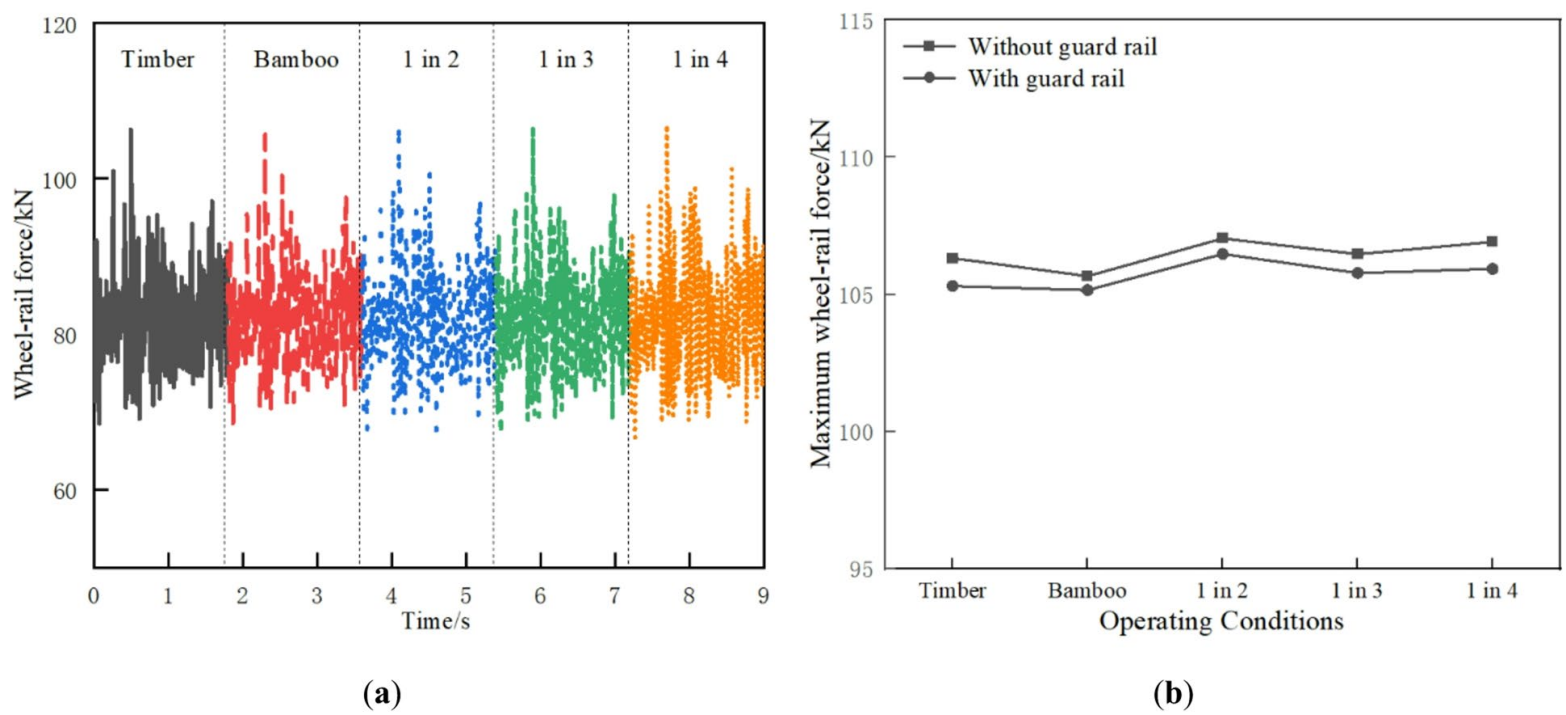
Wheel-rail force: (**a**) Time history curve; (**b**) Maximum value comparison.

The wheel-rail force under different operating conditions is shown in Fig. 11. As shown in Fig. 11a, the wheel-rail force increases when the timber sleepers are replaced in an interspersed manner due to the uneven stiffness of the track structure. The wheel load reduction rate can be calculated using the wheel-rail force. The wheel load reduction rates under different operating conditions are relatively small, all meeting the limit of 0.6 and demonstrating the safety of vehicle driving. Figure 11b reveals that the wheel-rail force is reduced with guard rail.

#### Rail acceleration

**Fig. 12:**
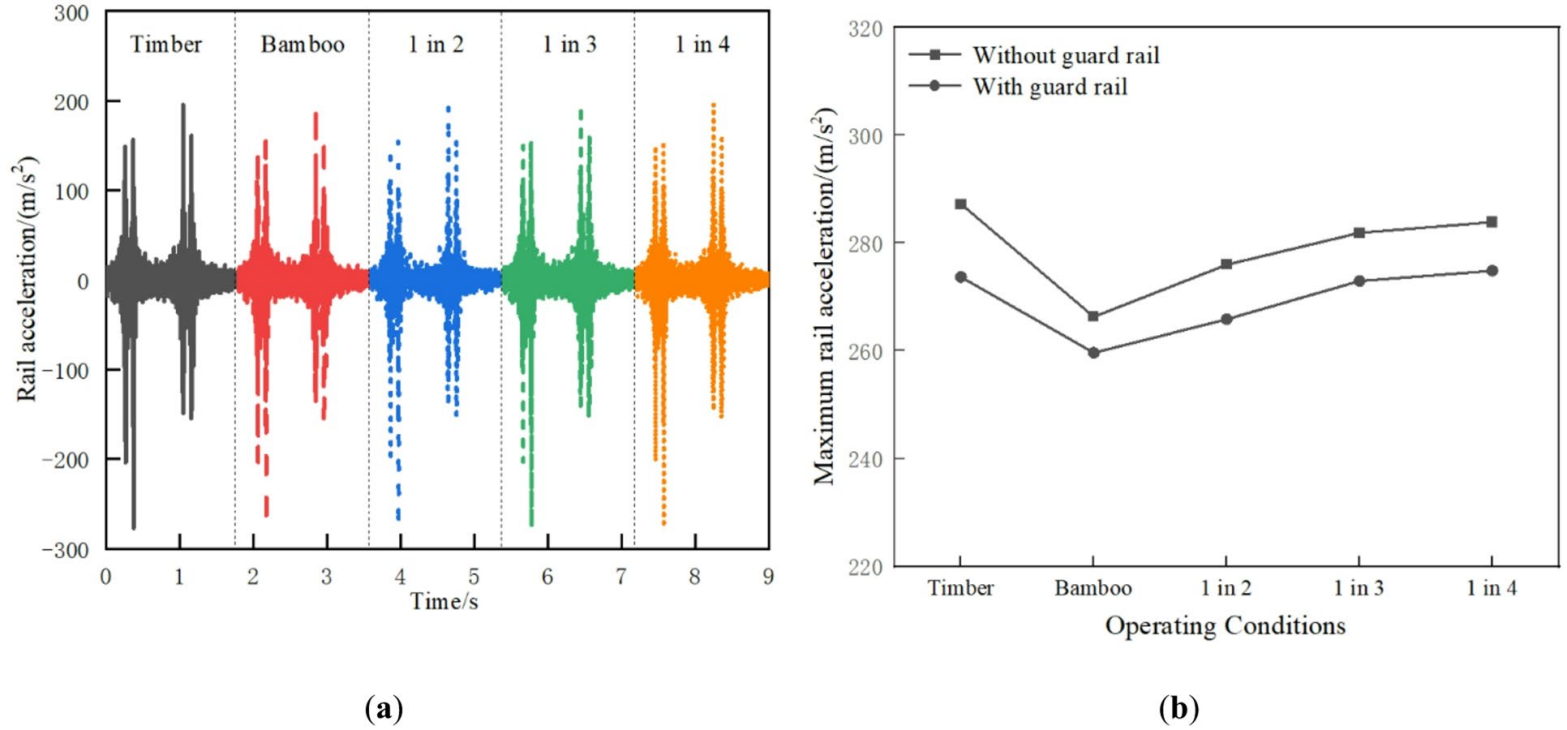
Rail acceleration: (**a**) Time history curve; (**b**) Maximum value comparison.

As shown in Fig. 12a, under different operating conditions, the rail acceleration of the all-timber sleepers track is slightly larger, at 287.23 m/s^2^. Compared with the all-timber sleepers track structure, the rail acceleration of 1 in 4, 1 in 3, 1 in 2 interspersed, and BCSs tracks decrease sequentially by approximately 1.17%, 1.86%, 3.92%, and 7.29%, respectively. The replacement of timber sleepers with BCSs is beneficial for reducing rail vibration. As shown in Fig. 12b, the rail acceleration is reduced with guard rail. This effect is most pronounced for timber sleepers.

#### Sleeper acceleration

**Fig. 13:**
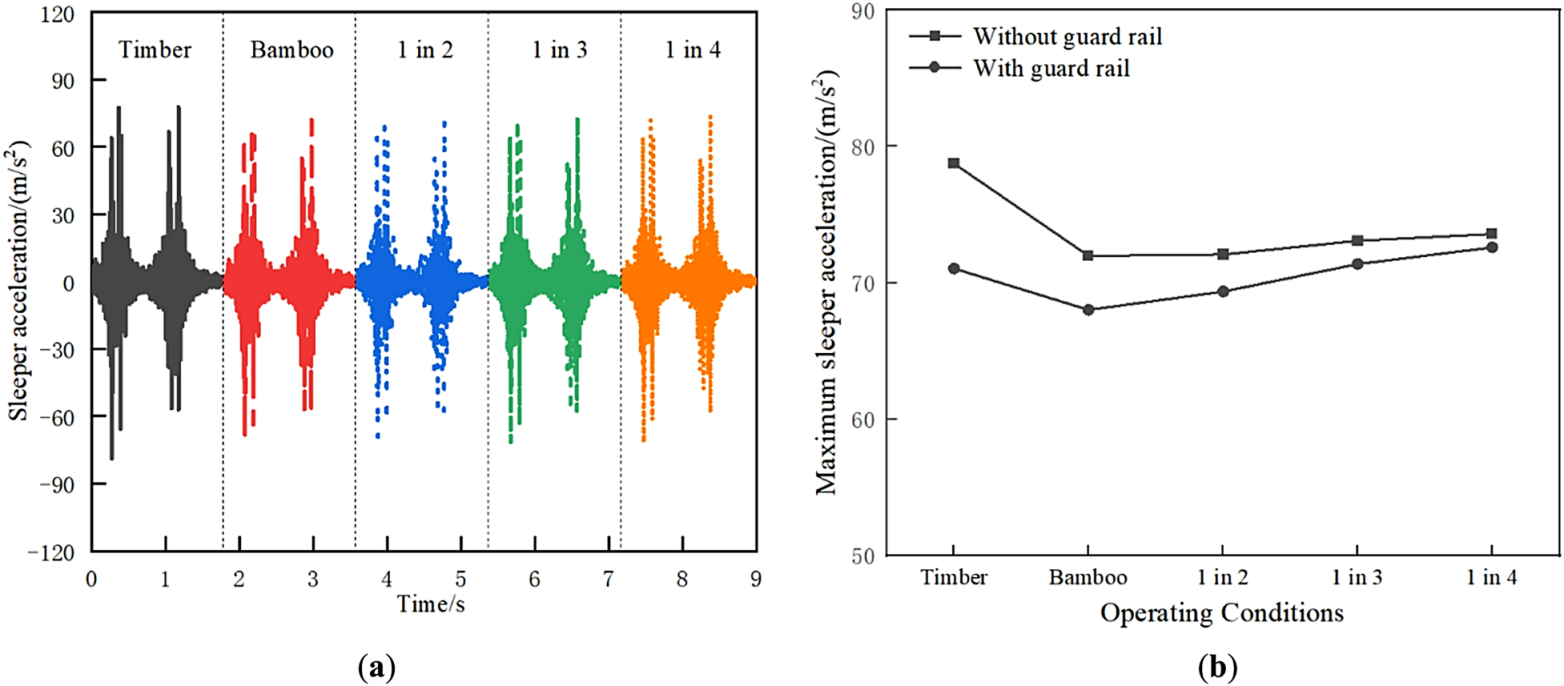
Sleeper acceleration: (**a**) Time history curve; (**b**) Maximum value comparison.

The sleeper acceleration is shown in Fig. 13. This pattern is similar to that of rail acceleration. The sleeper acceleration of the all-timber sleepers track is slightly larger, and the sleeper acceleration of 1 in 4, 1 in 3, 1 in 2, and BCSs tracks is reduced in sequence. The replacement of timber sleepers with BCSs is also beneficial for reducing sleeper vibration. From Fig. 13b, the sleeper acceleration is also reduced with guard rail; the effect is most pronounced for timber sleepers.

#### Bridge deck acceleration

**Fig. 14:**
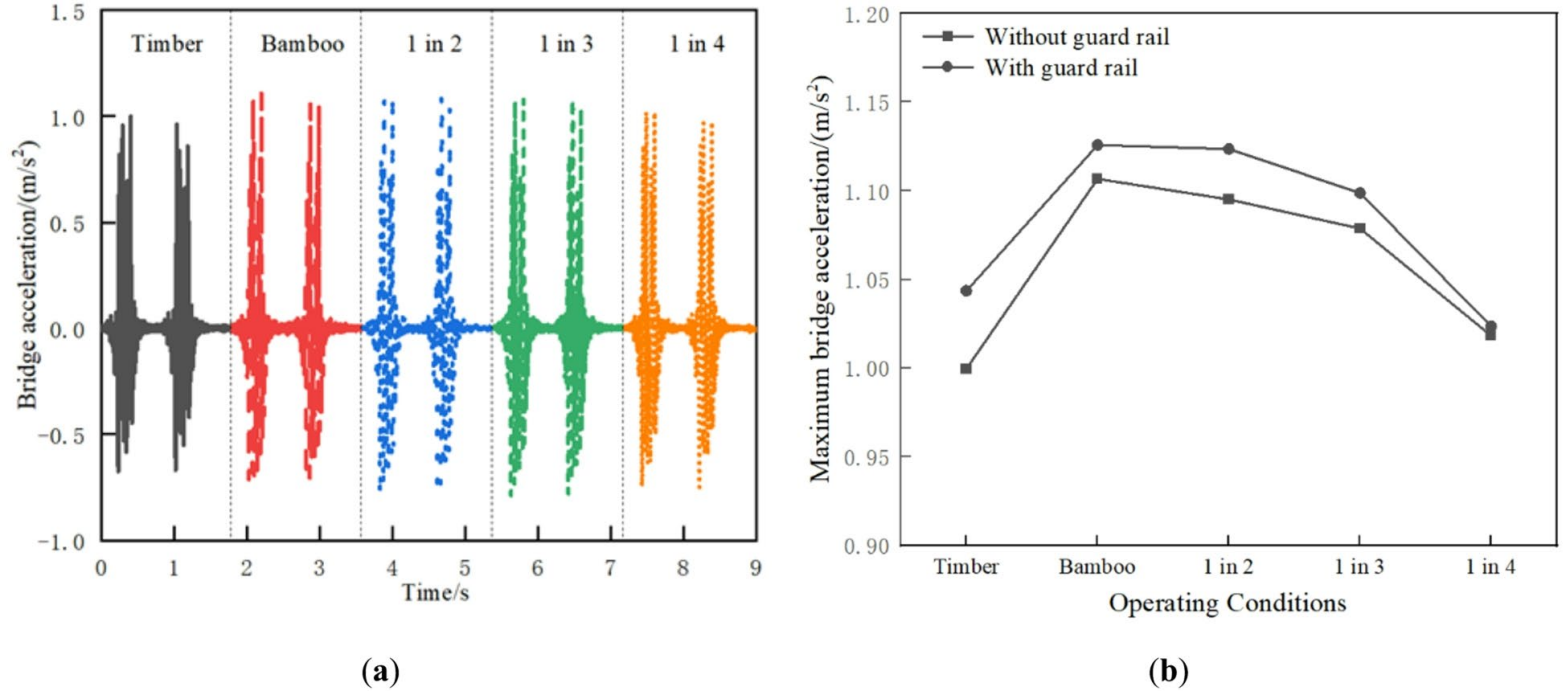
Bridge deck acceleration: (**a**) Time history curve; (**b**) Maximum value comparison.

As shown in Fig. 14a, the bridge deck acceleration of the all-timber sleepers track is the smallest, at approximately 0.99 m/s². Compared with the all-timber sleepers track, the bridge deck acceleration of 1 in 4, 1 in 3, 1 in 2, and BCSs tracks increase sequentially by approximately 1.85%, 7.90%, 9.54%, and 10.71%, respectively. Since the elastic modulus of BCSs is about twice that of timber sleepers, the replacement of timber sleepers with BCS increases the overall stiffness of the track, thus causing an increase in bridge deck vibration. The bridge deck vibration acceleration under different conditions is within the 3.5 m/s² limit specified in the “Code for Design on Railway Bridge and Culvert” (TB 10002 − 2017), thereby ensuring the safety of the bridge. From Fig. 14b, the bridge deck acceleration is increased with guard rail and the effect is most pronounced for timber sleepers.

### Normalization analysis

To more intuitively reflect the impact of different operating conditions on various responses, normalization method is used for the analysis. The normalization function is as follows:

3 $${\omega _i}=\frac{{{m_i}}}{{{m_1}+{m_2}+...+{m_n}}} \times 100\%$$

where, *i* corresponds to different operating conditions, ${\omega _i}$ represents the normalized function value of characteristic m under operating condition *i*, and *m*_*i*_ is the peak value of characteristic m under operating condition *i*, such as rail acceleration.

**Fig. 15:**
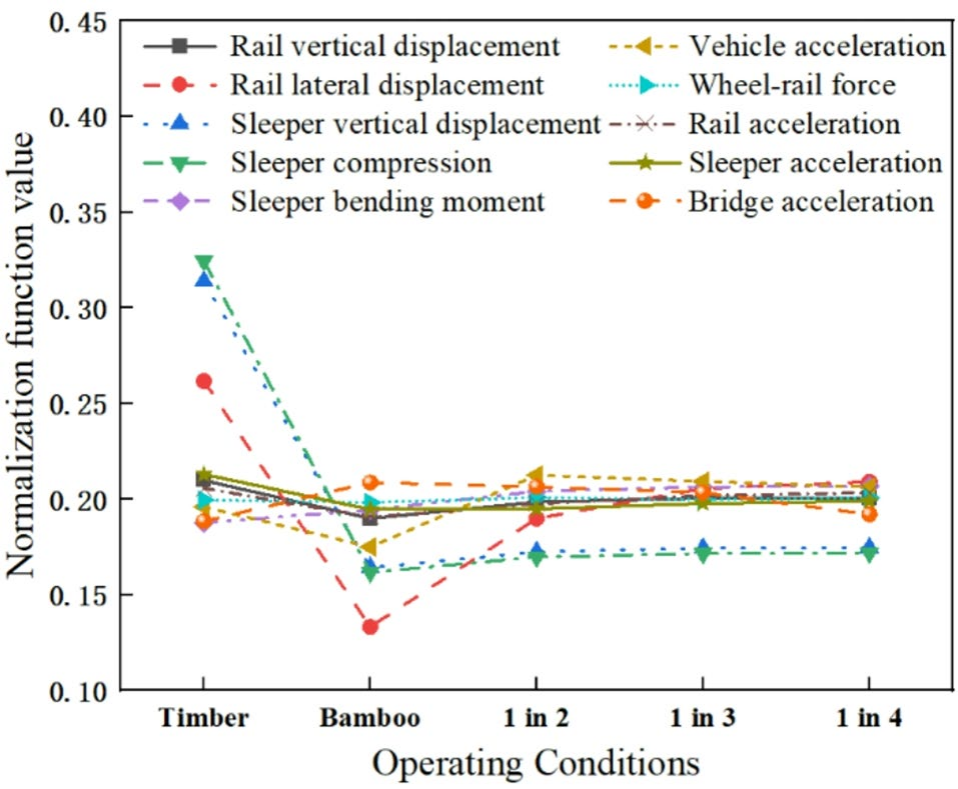
Normalization values of dynamic characteristics.

The normalized values of different responses are shown in Fig. 15. The figure shows that different levels of interspersed replacement of timber sleepers can significantly reduce sleeper compression, sleeper vertical displacement, and gauge reduction. Rail acceleration, sleeper acceleration, and rail vertical displacement are less sensitive to different levels of interspersed replacement of timber sleepers. The interspersed replacement of timber sleepers can increase the sleeper bending moment, vehicle acceleration, wheel-rail force, and bridge deck acceleration. The bending moment of BCSs is increased, but the tensile and compressive stresses remain below the allowable values, meaning BCSs meet the required strength parameters. The normalization analysis shows that the track structure maintains favorable mechanical properties after replacing timber sleepers with BCSs, though it slightly increases vehicle and bridge deck vibrations.

The differences in mechanical parameters between BCSs and timber sleepers lead to changes in the dynamic characteristics of each structural layer. Fortunately, the mechanical parameters of BCSs do not differ significantly from those of timber sleepers. Although vibrations of the vehicle body and bridge are increased, they remain well below safety limits, thereby ensuring structural safety. With continued train operation, timber sleepers gradually and randomly deteriorate. The maintenance strategy proposed is to gradually replace timber sleepers with BCSs in an interspersed manner, ultimately leading to complete replacement. Figure 15 shows that when all timber sleepers are replaced with BCSs, the track structure performs well in terms of maintaining track geometry and ensuring train safety. Therefore, for the long-term maintenance of timber sleepers on open bridge deck, the gradual replacement of timber sleepers with BCSs is feasible.

## Conclusions

This paper establishes a static model of the symmetrical open deck track and a vehicle-track coupling dynamic model, which were used to analyze the effect of the interspersed replacement timber sleepers with BCSs. The main conclusions are as follows:

1. After interspersed replacement of timber sleepers with BCSs, the sleeper compression, sleeper vertical displacement, and gauge reduction under the wheel load were reduced. The sleeper compression and sleeper vertical displacement decreased by over 40%. This is conducive to maintaining the track geometry, and the track gauge error is within the allowable range of 2 mm.
2. In terms of dynamic response, the interspersed replacement of timber sleepers with BCSs has little effect on rail acceleration, sleeper acceleration and the vertical displacement of rail; however, vehicle acceleration, wheel-rail force and bridge acceleration increase slightly, among which the vehicle acceleration for 1 in 2 interspersed track is the largest at 0.64 m/s², and the maximum bridge acceleration of the BCSs track is 1.13 m/s². The dynamic response of each structure is less than the limit.
3. Due to the compression and shear deformation in the sleeper cross section, the sleeper bending moment distribution differs from that of the four-point support beam. At the longitudinal beam support, the sleeper experiences compression at the bottom and tension at the top, resulting in a negative bending moment. Under different operating conditions, the maximum tensile and compressive stresses of the BCSs are 4.87 MPa and 7.97 MPa, both of which are below the allowable values, indicating the safety of using BCSs.
4. The analysis shows that the mechanical properties of the track structure are good after the interspersed replacement of timber sleepers with BCSs. The timber sleepers on the open bridge deck deteriorate randomly, and it is feasible to use BCSs for interspersed replacement. This study provides technical guidance for the replacement of timber sleepers on open bridge deck and a theoretical basis for the promotion and application of BCSs.

This study has limitations, including the complex practical application of replacing timber sleepers with interspersed BCSs on open bridge deck, the lack of consideration of lateral load transfer in the straight-line model, and an incomplete analysis of sensitivity factors under different conditions. Future research will expand to field testing and refine numerical models for various factors to expand the application scope of BCSs.

## Data Availability

The original contributions presented in this study are included in the article. Further inquiries can be directed to the corresponding author.
